# Supplementary material for: Anticancer Potential of Isoflavones: A Narrative Overview of Mechanistic Insights and Experimental Evidence from the Past Ten Years
Source: Biomedicines. 2025 Dec 5;13(12):2990. doi: 10.3390/biomedicines13122990 (PMC12730593; doi:10.3390/biomedicines13122990)
Supplement: Supplementary file 1 [file biomedicines-13-02990-s001.zip › biomedicines-4001974-supplementary.pdf]

Anticancer Potential of Isoflavones: A Narrative Overview of Mechanistic Insights and Experimental Evidence from the Past Ten Years

Maryna Schuenck Knupp [1], Lucas Nicolau de Queiroz [1], Mateus de Freitas Brito [2], Lucas Silva Abreu [2,*] and Bruno Kaufmann Robbs [3,*]

[1] Postgraduate Program in Applied Science for Health Products, Faculty of Pharmacy, Fluminense Federal University (UFF), Niteroi 24241-000, RJ, Brazil; marynask@id.uff.br (M.S.K.); lucasnicolaunf@gmail.com (L.N.Q.)

[2] Natural Products Chemistry Laboratory, Institute of Chemistry, Federal Fluminense University (UFF), Niteroi 24210-201, RJ, Brazil; mateusfb@id.uff.br (M.F.B.)

[3] Basic Science Department, Health Institute of Nova Friburgo, Fluminense Federal University (UFF), Nova Friburgo 28625-650, RJ, Brazil

* Correspondence: Correspondence: brunokr@id.uff.br (B.K.R.); abreu_lucas@id.uff.br (L.S.A.); +55 21 99772-1259 (B.K.R.); TEL.: +55 21 97719-0873 (L.S.A.)

SUPPLEMENTARY TABLE

Supplementary Tabel 1: List of compounds for which only a single study was identified in the literature, including the biological models used, observed antitumor activity and/or proposed mechanisms of action, and corresponding references. Cytotoxicity refers to general cell death with no mechanistic data available.

Compound	Animal/cell line tested	Antitumor activity and/or mechanism	Ref.
5,7-dihydroxy-4′-methoxy-6,8-diprenylisoflavone	U87MG.ΔEGFR (glioblastoma), MDA-MB-231 (breast carcinoma), CCRF-CEM cells (leukemia)	Cytotoxicity; ROS generation; Loss of mitochondrial membrane integrity; Cell cycle arrest (G0/G1 phase)	[1]
5,7-dimethoxy-6-(3 methyl-2-butenyl)-4'-hydroxyl isoflavones	HeLa (cervical adenocarcinoma)	Cytotoxicity	[2]
derrubone	KB (carcinoma epidermoid) and fibroblasts	Cytotoxicity; Loss of mitochondrial membrane potential (MMP)	[3]
erysubin B	HL-60 (leukemia)	Cytotoxicity; Apoptosis (caspase-dependent)	[4]
isoerythrin-A-4'-prenyl ether	A549 (lung carcinoma), LO2 (fetal hepatocyte)	Cytotoxicity	[5]
O6-BenzylglaziovianinA	HeLa (cervix adenocarcinoma)	Cell viability reduction; Cell cycle arrest; In vitro α/β-tubulin inhibition	[6]
3-(Benzo[b]thiophen-3-yl)-5,6,7-trimethoxy-4H-chromen-4-one	A549 (lung carcinoma), MDA-MB-231 and MCF-7 (breast adenocarcinoma) KB (epidermoid carcinoma), KB VIN (epidermoid carcinoma)	Cytotoxicity	[7]
3-(Naphthalen-1-yl)-4H-chromen-4-one (4)	None	Topoisomerase IIα inhibition	[8]
3',7-dihydroxy-4',8-dimethoxyisoflavone	MCF-7 (breast adenocarcinoma), TK10 (kidney carcinoma) and UACC62 (melanoma)	Cytotoxicity	[9]

7-((1-(4-fluorobenzyl)-1H-1,2,3-triazol-4-yl)methoxy)-3-(4-methoxyphenyl) 4H-chromen-4-one	A549 (lung carcinoma), Beas-2b (epithelial)	Cytotoxicity; Apoptosis; Downregulation of Bcl-2; Upregulation of Bax	[10]
7-acetyl-6,8-dimethoxy-4′-hydroxy-isoflavone	NB4 (human leukemia cell), A549 (lung carcinoma), SH-SY5Y (neuroblastoma), PC3 (prostate adenocarcinoma), MCF-7 (breast adenocarcinoma)	Cytotoxicity	[11]
Cytisine N-methylene-(7-hydroxy-4′ -methoxy)-isoflavone	A431 cells (carcinoma epidermoid), BT-474 (mamary gland ductal carcinoma) (over-express the EGFR and HER2 receptors) and MDA-MB-231 (breast adenocarcinoma) and 4T1 (mouse breast cancer stage 4)	Cytotoxicity; Dual EGFR/HER2 inhibition; Cell migration inhibition	[12]
ferrugone	A2780 and SKOV3 (ovary adenocarcinoma)	Apoptosis (caspase-dependent); Cytotoxicity; ROS production; Cell cycle arrest (sub-G1 phase)	[13]
Ficusaltin B	MDA-MB-231 and MCF-7 (breast adenocarcinoma), HepG2 (hepatocellular carcinoma) and Hacat (keratinocyte)	Cytotoxicity; Colony formation inhibition; Apoptosis	[14]
indicanine E	HL-60 (leukemia), SMMC-7721 (hepatocellular carcinoma), A-549 (lung carcinoma), MCF-7 (breast adenocarcinoma), and SW480 (colon adenocarcinoma)	Cytotoxicity	[15]
lupinalbin D	MDA-MB-231 and MCF-7 (breast adenocarcinoma), HepG2 (hepatocellular carcinoma) and Hacat (keratinocyte)	Cytotoxicity; Colony formation inhibition; Apoptosis	[14]

O7-Benzylglaziovianin A	HeLa (cervix adenocarcinoma)	Cell viability reduction; Cell cycle arrest; In vitro α/β-tubulin inhibition	[6]
Sophoricoside	U251 (glioma) and male BALB/c nude mice	Cytotoxicity; Colony formation inhibition; Xenograft tumor inhibition; AMPK upregulation; Cyclin D1, MYC and PCNA downregulation; Cell migration and invasion inhibition	[16]
8-prenylnaringenin	HCT116 (colon adenocarcinoma) and BV-2	Cytotoxicity; Nitric oxide production	[17]
(±) Erysectin A	Primary mouse microglial cells	Cytotoxicity	[18]
3′-formylalpinumisoflavone	HL-60 (leukemia), A-549 (lung carcinoma), SMMC-7721 (hepatocellular carcinoma), SW480 (colon adenocarcinoma)	Cytotoxicity	[15]
2-Phenyl-4H-chromen-4-one	HL-60 (leukemia), SMMC-7721 (hepatocellular carcinoma), A-549 (lung carcinoma) MCF-7 (breast adenocarcinoma), and SW480 (colon adenocarcinoma)	Cytotoxicity; Cell migration inhibition; Apoptosis; Colony formation inhibition; Tumor inhibition (xenograft); Upregulation of BAK1, P21, SUFU, BMP4	[19]
3-(2-fluorophenyl)-5-hydroxy-7-propoxy-4H chromen-4-one,	MDA-MB-231, MCF-7 (breast adenocarcinoma), and MDA-MB-435 (breast cancer cell line)	Cytotoxicity	[20]
3-(4-fluorophenyl)-7-hydroxy 2-(trifluoromethyl)-4H-chromen-4-one	A375 and A2058 (melanoma) and BALB/c nude mice	Cytotoxicity	[21]
3',4'-di-O-methylene-5-hydroxy-7-methoxy-6-isopentenyl isoflavone	MCF-7 (breast adenocarcinoma)	Cytotoxicity	[2]
3′-methyl tenuifone	HeLa (cervical adenocarcinoma)	Cytotoxicity; Apoptosis	[22]

3-Phenyl-4H-pyrido[1,2-a]pyrimidin-4-one	HL-60 (leukemia)	Cytotoxicity; PI3K/AKT/mTOR pathway inhibition	[23]
4′ -O-methylgrynullarin	PANC-1 (pancreatic carcinoma)	Inhibition of cell migration and colony formation under nutrient-rich conditions	[24]
4′,5′-tetramethoxyisoflavone, neoraunone	Colo-205 (colorectal adenocarcinoma) MCF-7 (breast adenocarcinoma) KB (epidermoid carcinoma), Vero (*Cercopithecus aethiops* kidney)	Cytotoxicity	[25]
4′-prenyloxyvigvexin A	U87MG.ΔEGFR cells (glioblastoma), CEM/ADR5000 cells (T-acute lymphoblastic leukemia)	Cytotoxicity	[26]
5,7,4′-trihydroxy-6,8-diprenylisoflavone	KB (epidermoid carcinoma), and NCI-H187 (lung carcinoma) and Vero (*Cercopithecus aethiops* kidney)	Cytotoxicity; Topoisomerase II catalytic inhibition	[27]
5-O-methyl-2′-methoxy-3′-methyl alpinumisoflavone	HL-60 (leukemia), SMMC-7721 (hepatocellular carcinoma), A-549 (lung carcinoma) MCF-7 (breast adenocarcinoma), and SW480 (colon adenocarcinoma)	Cytotoxicity	[15]
6-((2-(4-(2-(Dimethylamino)ethoxy)phenyl)-5-hydroxy-4-oxo[1]4H-chromen-7-yl)oxy)-N-hydroxyhexanamide	MDA-MB-231(breast adenocarcinoma) and 4T1 (mouse breast cancer stage 4)	Cytotoxicity; Cell cycle arrest (G1/S phase); Apoptosis; HDAC1/2/3/6 inhibition; STAT3 pathway inhibition	[28]
6-((2-Bromobenzyl)oxy)-3-(2,4,5-trimethoxyphenyl)-4H-chromen-4-one	HeLa (cervix adenocarcinoma), H460 (human large-cell lung carcinoma), HUVEC (primary human umbilical vein cells)	Inhibition of cancer cell proliferation; Tubulin polymerization inhibition; Cell migration inhibition; Cell cycle arrest (G2/M phase); Apoptosis (caspase-dependent); Anticancer activity (H460 xenograft); Cyclin B1 upregulation	[29]

6,7,3′-trimethoxy-4′,5′ methylenedioxyisoflavone	U87MG.ΔEGFR cells (glioblastoma), CEM/ADR5000 (T-acute lymphoblastic leukemia), Ramos (lymphoma), HCT 116 (adenocarcinoma colorectal), nude mice BALB/c	Cytotoxicity	[26]
6,7,4' - Trihydroxyisoflavone	KYSE 30, 450 and 510 (esophageal cancer), nude mice BALB/c	Pin1 isomerase inhibition; Cyclin D1 downregulation; Cell cycle arrest (G1/S phase); Apoptosis; Tumor growth inhibition (xenograft)	[30]
6,7-dimethoxy-3′,4′-methylenedioxy8-(3,3-dimethylallyl)isoflavone	A2780 and SKOV3 (ovary adenocarcinoma)	Apoptosis (caspase-dependent); Cytotoxicity; ROS production; Cell cycle arrest (sub-G1 phase)	[13]
6″,6″-Dimethylchromene-[2″,3″:7,8]-flavone	SW480 (colon adenocarcinoma)	Cytotoxicity	[31]
6-methoxy-4′ ,6′ -dimethylisoflavone-2′ ,5′ -quinone	TNBC (triple negative breast cancer)	Cytotoxicity; SIRT1 inhibition	[32]
7-((3-Methylbut-2-en-1-yl)oxy)-3-(2,4,5-trimethoxyphenyl)-4H-chromen-4-one	HUVEC (umbilical vein endothelial cells), HepG2 (hepatocellular carcinoma), A375 (melanoma), U251 (glioma), B16F10 (melanoma), and HCT116 cell (colorectal carcinoma) and zebra-fish angiogenesis model	Cell viability reduction; Antiangiogenic activity; Cell migration inhibition	[33]
7','3',4'- and 7',8',4'-trihydroxyisoflavone	HepG2 (hepatocellular carcinoma)	Cell viability reduction under hypoxic conditions	[34]
7,3′-dihydroxy-5′-methoxyisoflavone	MCF-7 (breast adenocarcinoma), TK10 (kidney carcinoma) and UACC62 (melanoma)	Cytotoxicity	[9]
7,4′-Bis-(2-hydroxy-3-tertbutylaminopropoxy]isoflavone	MDA-MB-231 (breast adenocarcinoma), HT-29 (colon carcinoma), HCT116 (colon adenocarcinoma),	Cytotoxicity; Nrf2/ARE pathway activation	[35]

	HepG2 (hepatocellular carcinoma)		
7-acetyl-4′,6-dimethoxy-isoflavone,	NB4 (human leukemia cell), A549 (lung carcinoma), SH-SY5Y (neuroblastoma), PC3 (prostate adenocarcinoma), MCF-7 (breast adenocarcinoma)	Cytotoxicity	[11]
7-acetyl-4′-hydroxy-6-methoxy-isoflavone	NB4 (human leukemia cell), A549 (lung carcinoma), SH-SY5Y (neuroblastoma), PC3 (prostate adenocarcinoma), MCF-7 (breast adenocarcinoma)	Cytotoxicity	[11]
7-O-carboxymethyl 4′-fluoro-2-trifluormethylisoflavone	MCF-7 (breast adenocarcinoma)	Cytotoxicity	[21]
7-O-carboxymethyl-4′-fluoroisoflavone	MCF-7 (breast adenocarcinoma)	Cytotoxicity	[21]
8-prenylnarigenin	HCT116 (colon adenocarcinoma)	Cytotoxicity	[17]
Auriculasin	NSCLC cells (non-small cell lung cancer) and nude mice BALB/c	Cytotoxicity; Mitochondrial oxidative stress; Ferroptosis (↑ACSL4, ↑PTGS2, ↓FSP1, ↓GPX4); ROS generation; Tumor inhibition (xenograft); PI3K/Akt pathway inhibition	[36]
Cytisine N-methylene-(4′ ,5,7-trihydroxy)-isoflavone	A431 cells (carcinoma epidermoid) and BT-474 (mammary gland ductal carcinoma) (over-express the EGFR and HER2 receptors) and MDA-MB-231 (breast adenocarcinoma) and 4T1 (mouse breast cancer stage 4)	Cytotoxicity; Dual EGFR/HER2 inhibition; Cell migration inhibition	[12]
Erythraddison A	HL-60 (leukemia), SMMC-7721 (hepatocellular carcinoma), A-549 (lung carcinoma), MCF-7 (breast	Cytotoxicity	[15]

	adenocarcinoma), and SW480 (colon adenocarcinoma)		
Glaziovianin A	HeLa (cervix adenocarcinoma)	Cell viability reduction; Cell cycle arrest; In vitro α/β-tubulin inhibition	[6]
glyurallin	KB (epidermoid carcinoma) and fibroblasts	Cytotoxicity; Loss of mitochondrial membrane potential (MMP)	[3]
lupalbigenin	KB (epidermoid carcinoma), NCI-H187 (lung carcinoma) and Vero (*Cercopithecus aethiops* kidney)	Cytotoxicity; Topoisomerase II catalytic inhibition	[27]
mappianthone A	HL-60 (leukemia), SMMC-7721 (hepatocellular carcinoma), A-549 (lung carcinoma), MCF-7 (breast adenocarcinoma), and SW480 (colon adenocarcinoma)	Cytotoxicity	[15]
methyl 4-([1,1'-biphenyl]-4-yl)-2,2-difluoro-4-(4-oxo-3-phenyl-4H-chromen-2-yl)butanoate	MCF-7 and MDA-MB-231 (breast adenocarcinoma)	Cytotoxicity; Cell migration inhibition; MMP-2 inhibition	[37]
osajin	U87MG.ΔEGFR (glioblastoma), MDA-MB-231 (breast carcinoma) CCRF-CEM (leukemia)	Cytotoxicity; ROS generation; Loss of mitochondrial membrane integrity; Cell cycle arrest (G0/G1 phase)	[1]
pumilaisoflavone D	HL-60 (leukemia), SMMC-7721 (carcinoma hepatocelular), A-549 (lung carcinoma), MCF-7 (breast adenocarcinoma), and SW480 (colon adenocarcinoma)	Cytotoxicity	[15]
rhoifolin-7-O-β-D-neohesperidoside	EACC (Ehrlich ascites carcinoma)	Cytotoxicity	[38]

References:

[1] F. A. Adem *et al.*, "Cytotoxicity of isoflavones and biflavonoids from Ormocarpum kirkii towards multi-factorial drug resistant cancer," *Phytomedicine*, vol. 58, May 2019, doi: 10.1016/j.phymed.2019.152853.

[2] L. F. Yang *et al.*, "Isolation and characterization of a new bioactive isoflavone from Derris eriocarpa," *J Asian Nat Prod Res*, vol. 17, no. 10, pp. 1002–1009, Aug. 2015, doi: 10.1080/10286020.2015.1042370.

[3] C. Ito *et al.*, "Three isoflavones from Derris scandens (Roxb.) Benth and their cancer chemopreventive activity and in vitro antiproliferative effects," *Phytochemistry*, vol. 175, Jul. 2020, doi: 10.1016/j.phytochem.2020.112376.

[4] K. Hikita *et al.*, "Inhibitory Effect of Isoflavones from Erythrina poeppigiana on the Growth of HL-60 Human Leukemia Cells through Inhibition of Glyoxalase I."

[5] D. Buyinza *et al.*, "Cytotoxicity of isoflavones from Millettia dura," *Nat Prod Res*, vol. 35, no. 16, pp. 2744–2747, 2021, doi: 10.1080/14786419.2019.1660335.

[6] I. Hayakawa *et al.*, "Discovery of O6-benzyl glaziovianin A, a potent cytotoxic substance and a potent inhibitor of α,β-tubulin polymerization," *Bioorg Med Chem*, vol. 24, no. 21, pp. 5639–5645, 2016, doi: 10.1016/j.bmc.2016.09.026.

[7] S. Hirazawa, Y. Saito, M. Sagano, M. Goto, and K. Nakagawa-Goto, "Chemical Space Expansion of Flavonoids: Induction of Mitotic Inhibition by Replacing Ring B with a 10π-Electron System, Benzo[b]thiophene," Jan. 28, 2022, *American Chemical Society*. doi: 10.1021/acs.jnatprod.1c00867.

[8] G. Priyadarshani, S. Amrutkar, A. Nayak, U. C. Banerjee, C. N. Kundu, and S. K. Guchhait, "Scaffold-hopping of bioactive flavonoids: Discovery of aryl-pyridopyrimidinones as potent anticancer agents that inhibit catalytic role of topoisomerase IIα," *Eur J Med Chem*, vol. 122, pp. 43–54, 2016, doi: 10.1016/j.ejmech.2016.06.024.

[9] E. Korir, J. J. Kiplimo, N. R. Crouch, N. Moodley, and N. A. Koorbanally, "Isoflavones from Calpurnia aurea subsp. aurea and their anticancer activity," *African Journal of Traditional, Complementary and Alternative Medicines*, vol. 11, no. 5, pp. 33–37, 2014, doi: 10.4314/ajtcam.v11i5.5.

[10] W. D. Jia *et al.*, “Synthesis, molecular docking studies of formononetin derivatives as potent Bax agonists for anticancer activity,” *Nat Prod Res*, vol. 39, no. 3, pp. 423–437, 2025, doi: 10.1080/14786419.2023.2269592.

[11] T. Cui *et al.*, “Three new isoflavones from the Pueraria montana var. lobata (Willd.) and their bioactivities,” *Nat Prod Res*, vol. 32, no. 23, pp. 2817–2824, Dec. 2018, doi: 10.1080/14786419.2017.1385008.

[12] Y. Wang, X. Yin, L. Chen, Z. Yin, and Z. Zuo, “Discovery and evaluation of cytisine N-isoflavones as novel EGFR/HER2 dual inhibitors,” *Bioorg Chem*, vol. 127, Oct. 2022, doi: 10.1016/j.bioorg.2022.105868.

[13] Y. Y. Wang, J. H. Kwak, K. T. Lee, T. Deyou, Y. P. Jang, and J. H. Choi, “Isoflavones isolated from the seeds of Millettia ferruginea induced apoptotic cell death in human ovarian cancer cells,” *Molecules*, vol. 25, no. 1, Jan. 2020, doi: 10.3390/molecules25010207.

[14] J. Yao, Z. Wang, R. Wang, Y. Wang, J. Xu, and X. He, “Anti-proliferative and anti-inflammatory prenylated isoflavones and coumaronochromones from the fruits of Ficus altissima,” *Bioorg Chem*, vol. 113, Aug. 2021, doi: 10.1016/j.bioorg.2021.104996.

[15] Y. P. Liu, L. L. Sun, X. L. Zhang, H. Y. Niu, Z. H. Pan, and Y. H. Fu, “Prenylated isoflavones with potential antiproliferative activities from Mappianthus iodoides,” *Nat Prod Res*, vol. 34, no. 16, pp. 2295–2300, Aug. 2020, doi: 10.1080/14786419.2018.1536132.

[16] C. Wang *et al.*, “Sophoricoside Inhibited Glioblastoma Cell Progression Through Activated AMP-Activated Protein Kinase (AMPK),” *Mol Carcinog*, vol. 64, no. 5, pp. 816–828, May 2025, doi: 10.1002/mc.23889.

[17] C. Y. Liu *et al.*, “Coumaronochromones, flavanones, and isoflavones from the twigs and leaves of Erythrina subumbrans inhibit PTP1B and nitric oxide production,” *Phytochemistry*, vol. 206, Feb. 2023, doi: 10.1016/j.phytochem.2022.113550.

[18] R. Luo, F. Li, H. Zhuang, and L. Wang, “(±) Erysectin A, a new isoprenylated isoflavone with a rare acetonyl group from Erythrina secundiflora Hassk,” *Nat Prod Res*, vol. 36, no. 19, pp. 4886–4891, 2022, doi: 10.1080/14786419.2021.1908280.

[19] C. Liang *et al.*, “The anti-melanoma roles and mechanisms of tricholoma isoflavone derivative CA028,” *NPJ Sci Food*, vol. 9, no. 1, Dec. 2025, doi: 10.1038/s41538-025-00370-6.

[20] Z. Yang *et al.*, "Design, Synthesis, and Anti-Breast Cancer Activity of Novel Fluorinated 7-O-Modified Genistein Derivatives," *Med Chem (Los Angeles)*, vol. 19, no. 1, pp. 64–74, Jun. 2022, doi: 10.2174/1573406418666220607140651.

[21] M. S. Ayoup *et al.*, "Efficient Consecutive Synthesis of Fluorinated Isoflavone Analogs, X-Ray Structures, Hirshfeld Analysis, and Anticancer Activity Assessment," *Molecules*, vol. 30, no. 4, Feb. 2025, doi: 10.3390/molecules30040795.

[22] M. A. Al-Qudah *et al.*, "New isoflavones from Gynandriris sisyrinchium and their antioxidant and cytotoxic activities," *Fitoterapia*, vol. 107, pp. 15–21, Dec. 2015, doi: 10.1016/j.fitote.2015.09.020.

[23] B. Lawal *et al.*, "A preclinical report of a cobimetinib-inspired novel anticancer small-molecule scaffold of isoflavones, NSC777213, for targeting PI3K/AKT/mTOR/MEK in multiple cancers," 2021. [Online]. Available: www.ajcr.us/

[24] S. Sun *et al.*, "A new anti-austerity agent, 4′-O-methylgrynullarin from Derris scandens induces PANC-1 human pancreatic cancer cell death under nutrition starvation via inhibition of Akt/mTOR pathway," *Bioorg Med Chem Lett*, vol. 40, May 2021, doi: 10.1016/j.bmcl.2021.127967.

[25] S. Singha, J. Yahuafai, and S. Sutthivaiyakit, "12-Nor-rotenoids and other cytotoxic constituents of Pachyrhizus erosus seeds," *Phytochemistry*, vol. 229, Jan. 2025, doi: 10.1016/j.phytochem.2024.114281.

[26] F. A. Adem *et al.*, "Cytotoxic flavonoids from two Lonchocarpus species," *Nat Prod Res*, vol. 33, no. 18, pp. 2609–2617, Sep. 2019, doi: 10.1080/14786419.2018.1462179.

[27] S. Sangmalee, A. Laorpaksa, B. Sritularak, and S. Sukrong, "Bioassay-Guided Isolation of Two Flavonoids from Derris scandens with Topoisomerase II Poison Activity," 2016.

[28] M. Wei *et al.*, "Design and synthesis of novel Flavone-based histone deacetylase inhibitors antagonizing activation of STAT3 in breast cancer," *Eur J Med Chem*, vol. 206, Nov. 2020, doi: 10.1016/j.ejmech.2020.112677.

[29] W. Yan *et al.*, "Crystal structure of tubulin-barbigerone complex enables rational design of potent anticancer agents with isoflavone skeleton," *Phytomedicine*, vol. 109, Jan. 2023, doi: 10.1016/j.phymed.2022.154550.

[30] T. G. Lim *et al.*, "The prolyl isomerase pin1 is a novel target of 6,7,40-trihydroxyisoflavone for suppressing esophageal cancer growth," *Cancer Prevention Research*, vol. 10, no. 5, pp. 308–318, May 2017, doi: 10.1158/1940-6207.CAPR-16-0318.

[31] P. Meesakul *et al.*, "Rotenoids and isoflavones from the leaf and pod extracts of Millettia brandisiana Kurz," *Phytochemistry*, vol. 204, Dec. 2022, doi: 10.1016/j.phytochem.2022.113440.

[32] M. A. Selepe *et al.*, "Synthesis and evaluation of benzoylbenzofurans and isoflavone derivatives as sirtuin 1 inhibitors with antiproliferative effects on cancer cells," *Bioorg Chem*, vol. 128, Nov. 2022, doi: 10.1016/j.bioorg.2022.106101.

[33] G. Wang *et al.*, "Synthesis, structure-activity relationships and biological evaluation of barbigerone analogues as anti-proliferative and anti-angiogenesis agents," *Bioorg Med Chem Lett*, vol. 24, no. 14, pp. 3158–3163, Jul. 2014, doi: 10.1016/j.bmcl.2014.04.121.

[34] W. S. Tzeng, W. L. Teng, P. H. Huang, F. L. Yen, and Y. L. Shiue, "Anti-cancer activity and cellular uptake of 7,3′,4′- and 7,8,4′-trihydroxyisoflavone in HepG2 cells under hypoxic conditions," *J Enzyme Inhib Med Chem*, vol. 39, no. 1, 2024, doi: 10.1080/14756366.2023.2288806.

[35] J.-J. Tang, G. Xiao-Ting, W. Ya-Jing, Z. Tian-Yu, L. U. Jin-Rong, and H. U. Rong, "Chinese Journal of Natural Medicines Synthesis and cytotoxicity evaluation of 3-amino-2-hydroxypropoxyisoflavone derivatives," 2016.

[36] X. Wang, T. Zhang, L. Qu, Y. Zhang, and G. Gao, "Auriculasin induces mitochondrial oxidative stress and drives ferroptosis by inhibiting PI3K/Akt pathway in non-small cell lung cancer," *Naunyn Schmiedebergs Arch Pharmacol*, vol. 398, no. 1, pp. 967–977, Jan. 2025, doi: 10.1007/s00210-024-03328-9.

[37] Y. Q. Liu *et al.*, "N-Heterocyclic Carbene Organocatalysis Enabled Modular Synthesis of Fluorinated Isoflavonoids to Suppress Proliferation and Migration in Breast Cancer Cells," *Advanced Science*, vol. 12, no. 13, Apr. 2025, doi: 10.1002/advs.202413851.

[38] M. Marzouk, S. M. Khalifa, A. H. Ahmed, A. M. Metwaly, H. Sh. Mohammed, and H. A. A. Taie, "LC/HRESI-MS/MS screening, phytochemical characterization, and in vitro antioxidant and cytotoxic potential of Jatropha integerrima Jacq. extracts," *Bioorg Chem*, vol. 140, Nov. 2023, doi: 10.1016/j.bioorg.2023.106825.
